# Targeted Next Generation Sequencing Identifies Novel Mutations in *RP1* as a Relatively Common Cause of Autosomal Recessive Rod-Cone Dystrophy

**DOI:** 10.1155/2015/485624

**Published:** 2015-01-06

**Authors:** Said El Shamieh, Elise Boulanger-Scemama, Marie-Elise Lancelot, Aline Antonio, Vanessa Démontant, Christel Condroyer, Mélanie Letexier, Jean-Paul Saraiva, Saddek Mohand-Saïd, José-Alain Sahel, Isabelle Audo, Christina Zeitz

**Affiliations:** ^1^INSERM, U968, 75012 Paris, France; ^2^Sorbonne Universités, UPMC University, Paris 06, UMR_S 968, Institut de la Vision, 75012 Paris, France; ^3^CNRS, UMR_7210, 75012 Paris, France; ^4^IntegraGen SA, Genopole Campus 1, Building G8, 91030 Evry, France; ^5^Centre Hospitalier National d'Ophtalmologie des Quinze-Vingts, DHU ViewMaintain, INSERM-DHOS CIC 1423, 75012 Paris, France; ^6^Fondation Ophtalmologique Adolphe de Rothschild, 75019 Paris, France; ^7^Académie des Sciences-Institut de France, 75006 Paris, France; ^8^University College London Institute of Ophthalmology, 11-43 Bath Street, London EC1V 9EL, UK

## Abstract

We report ophthalmic and genetic findings in families with autosomal recessive rod-cone dystrophy (arRCD) and *RP1* mutations. Detailed ophthalmic examination was performed in 242 sporadic and arRCD subjects. Genomic DNA was investigated using our customized next generation sequencing panel targeting up to 123 genes implicated in inherited retinal disorders. Stringent filtering coupled with Sanger sequencing and followed by cosegregation analysis was performed to confirm biallelism and the implication of the most likely disease causing variants. Sequencing identified 9 *RP1* mutations in 7 index cases. Eight of the mutations were novel, and all cosegregated with severe arRCD phenotype, found associated with additional macular changes. Among the identified mutations, 4 belong to a region, previously associated with arRCD, and 5 others in a region previously associated with adRCD. Our prevalence studies showed that *RP1* mutations account for up to 2.5% of arRCD. These results point out for the necessity of sequencing *RP1* when genetically investigating sporadic and arRCD. It further highlights the interest of unbiased sequencing technique, which allows investigating the implication of the same gene in different modes of inheritance. Finally, it reports that different regions of *RP1* can also lead to arRCD.

## 1. Introduction

Rod-cone dystrophy (RCD), also known as retinitis pigmentosa, is a heterogeneous group of inherited disorders affecting primary rod photoreceptors in the majority of cases with secondary cone degeneration [1, 2]. Population-based studies showed that 1 in 4,000 individuals is affected around the world [1]. Patients diagnosed with RCD initially complain of night blindness due to rod dysfunction followed by progressive visual field constriction, abnormal color vision, and eventually loss of central vision due to cone photoreceptor involvement [1].

RCD is inherited as a Mendelian trait in most cases [3]. On the basis of its mode of inheritance and prevalence, RCD can be divided into 3 groups: autosomal dominant (ad) (30–40%), autosomal recessive (ar) (50–60%), and X-linked (xl) (5–15%) [3]. To date, mutations in at least 53 genes were reported to cause nonsyndromic RCD (till 25 June 2014, https://sph.uth.edu/retnet/). Prevalence studies revealed* rhodopsin* (*RHO*),* retinitis pigmentosa GTPase regulator* (*RPGR*), and* usherin* (*USH2A*) as being the most frequently mutated genes in adRCD [4, 5], xlRCD [4], and arRCD, respectively [6]. Of note is that many other genes with lower prevalence are also implicated in the genetic etiology of RCD [7, 8]. Mutations in* RP1* were first shown to cause adRCD [9–11]; however, since 2005, articles have shed light on its implication in arRCD etiology [12–20].* RP1* mutations were shown to account for ≈5.5% and ≈1% of adRCD and arRCD cases, respectively [8–20]. Interestingly, Avila-Fernandez et al. [12] reported that a founder nonsense mutation in the Spanish population p.Ser542^*^ is responsible for 4.5% of arRCD cases suggesting that* RP1* mutations are more prevalent in arRCD than previously thought [12].

Retinitis pigmentosa 1 (*RP1*) is a photoreceptor-specific gene encoding a protein regulated by oxygen [10]. RP1 protein is required for correct orientation and higher-order stacking of outer segment disks [21] and was shown to be part of the photoreceptor axoneme [22]. RP1 localizes to the connecting cilia of photoreceptors and may assist in maintenance of ciliary structure or transport down the photoreceptor [22]. Like many retinal degeneration genes, the mechanism by which mutations in* RP1* lead to photoreceptor cell death is still unclear.

We developed an unbiased and time-efficient retinal gene next generation sequencing array (NGS), which was further revised and improved to target more than 120 genes implicated in inherited retinal diseases (IRDs) (list available upon request) [23]. Using this NGS panel, we screened a total of 242 subjects with sporadic and recessive RCD in order to detect disease causing mutations and to report the prevalence of pathogenic mutations in* RP1* causing arRCD.

## 2. Methods

### 2.1. Ethics Statement and Clinical Diagnosis of Rod-Cone Dystrophy

The study protocol adhered to the tenets of the Declaration of Helsinki and was approved by the local Ethics Committee (CPP, Ile de France V). Informed written consent was obtained from each study participant. Index patients underwent full ophthalmic examination as previously described [23].

### 2.2. Targeted Next Generation Sequencing

A cohort of 242 subjects affected with sporadic and arRCD was investigated in the present study. Prior to NGS screening, molecular genetic analysis with microarray (Asper Ophthalmics, Tartu, Estonia), followed by direct Sanger sequencing of* EYS* and* C2orf71* (major and minor genes implicated in RCD, newly discovered at the beginning of our study), was performed in 201 index subjects (82%) [2, 24]. As* RPGR* exon* ORF15* (MIM 312610) is not targeted by existing NGS panels, we excluded mutations in this “hot spot” by Sanger sequencing. Although our NGS panel was selected from the SureSelect Human All Exon Kits Version 4 (Agilent, Massy, France), this design was improved after analyzing the first 83 subjects with sporadic and arRCD. More precisely, a total of ≈300 Kb regions were added in order to cover all the previously nontargeted regions. Thus, whereas the first design covered the exons and the flanking intronic regions of 120 genes implicated in IRDs, the second covered 123 genes in total. The eArray web-based probe design tool was used for this purpose (https://earray.chem.agilent.com/earray). All probes were designed and synthesized by Agilent Technologies (Santa Clara, CA, USA). Sequence capture, enrichment, and elution were performed according to Agilent's instructions. The complete details were described elsewhere [23].

### 2.3. Assembly and Variant Calling

Sequence reads were aligned to the reference human genome (UCSC hg19) using CASAVA1.7 software (Illumina) and the ELANDv2 alignment algorithm. Sequence variation annotation was performed using the IntegraGen in-house pipeline, which consisted of gene annotation (RefSeq), detection of known single nucleotide polymorphisms (dbSNP 135) followed by mutation characterization (missense, intronic, synonymous, nonsense, splice site, and insertions/deletions).

### 2.4. Quality Control and Coverage Assessment

The first NGS retinal panel harbored 120 IRDs genes, encompassing 321,240 kb length per sample. However, after improvement, the same panel contained ≈600 Kb and covered 123 IRD genes. The depth of coverage was calculated by counting the number of sequenced bases mapping to the target regions. Mean depth of coverage was calculated per base pair for all samples; however, only the results of subjects having* RP1* mutations were shown.

### 2.5. Discrete Filtering of Annotated Variants

In order to identify disease causing mutations among nonpathogenic single nucleotide polymorphisms, we used a filtering approach against a set of polymorphisms that are available in the public databases: dbSNP 137, 1000 Genomes Project [25], HapMap [26], and Exome Variant Server [27] with removal of variants with a minor allele frequency (MAF) ≥ 0.005 in case of presumed autosomal recessive mode of inheritance.

### 2.6. Pathogenicity Assessment

We stratified candidate mutations based on their functional class by giving a priority to frameshifts, stop codons, and disruptions of canonical splice sites variants [28]. For missense changes, amino acid conservation across 46 different species was studied using the UCSC Genome Browser [29]. If no amino acid change was found, then the residue was considered as “highly conserved.” If a different change was seen in less than four species and not in the primates, then it was considered as “moderately conserved” and if a change was present in 5–7, it was considered as “marginally conserved”; otherwise, the amino acid residue was considered as “not conserved.” Pathogenic prediction was performed using two software programs: Polyphen2 [30] and SIFT [31], based on species/homologue conservation, putative structural domains, and 3D structures (if available). Analysis of potential splice site variant consequences when relevant was done using human splicing finder [32].

### 2.7. Known Genotype-Phenotype Correlations

The search for previous genotype-phenotype associations was done by searching numerous literature databases, including Online Mendelian Inheritance in Man (http://omim.org/), Human Gene Mutation Database [33], Leiden Open Variation Database [34], and RetNet (https://sph.uth.edu/retnet/).

### 2.8. Validation of the Identified Variants and Cosegregation Analyses

Sanger sequencing was performed to validate disease causing mutations in* RP1*. The respective primer information can be communicated on request. In addition, blood samples were collected from additional family members when possible and cosegregation analyses on extracted DNA were performed as previously described [35, 36].

## 3. Results

### 3.1. Clinical Data

**Figure 1:**
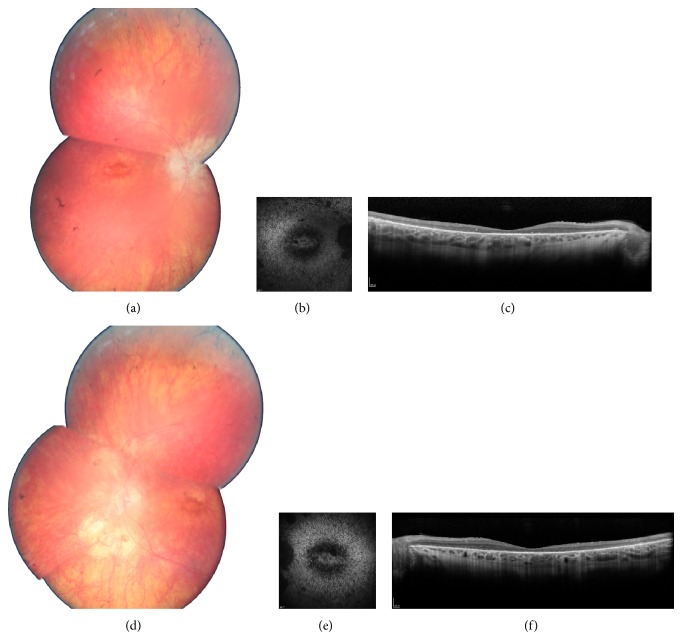
Ophthalmic features of family F752: II.1 (CIC01245): fundus color photographs ((a) and (d) for right and left eye resp.), autofluorescence ((b) and (e) for right and left eye resp.), and spectral domain optical coherence tomography horizontal macula scans ((c) and (f) for right and left eye resp.), showing severe rod-cone dystrophy signs with macular involvement.

Clinical data are summarized in Table 1. Among identified patients, 5 were females, 2 were male, and ages at time of examination ranged from 25 to 42. All patients were diagnosed before age 20 mostly based on night blindness from early childhood and secondary central vision loss. They all showed severe RCD with constricted visual fields, no detectable responses on full field electroretinogram, and both peripheral involvement and macular involvement (Figure 1 presents fundus pictures of patient II.1 (CIC01245) in family F752 as an example). Comparing visual acuity and visual fields for these arRCD patients with those of adRCD cases published by Audo and coworkers [8], we noticed a more severe phenotype in recessive cases. However, more cases with* RP1* mutations would be needed to draw statistical conclusion.

### 3.2. Sequencing Statistics

**Figure 2:**
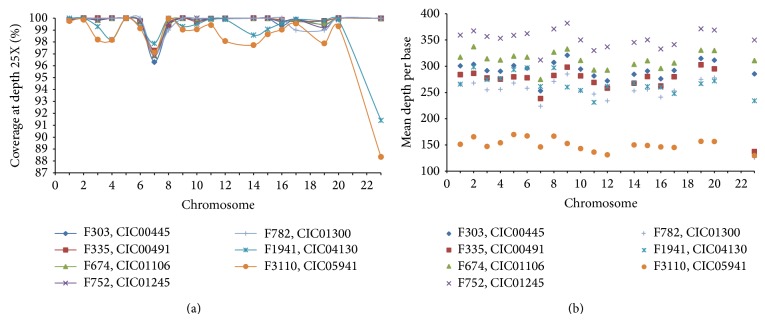
Sequencing statistics in index patients. (a) The overall sequencing coverage of the target regions at 25X depth of coverage is shown in each of the chromosomes. No values were indicated for chromosomes 13, 18, 21, and 22 as they were not targeted. The term chromosome 23 was used to designate the X chromosome. F1941: III.1** (**CIC04130) and F3110: III.5 (CIC05941) showed the lowest coverage results. (b) The average mean depth per base pair is shown for each of the chromosomes. Most targets showed coverage around 300 times.

In index patients, the overall sequencing coverage of the target regions was ≥88% for a 25X depth of coverage in each of the chromosomes (Figure 2(a)), resulting a mean sequencing depth of 299 times per base. Mean sequencing results per base in each target chromosome gene regions were shown in Figure 2(b). It is of importance to mention that <1% of target regions were not covered at all. These were fragments of 120 bp belonging in 66% of the cases only to a fraction of an exon. The remaining uncovered targets corresponded each to an entire exon in genes such as* CHM*,* PDZD7*,* RP9*,* CC2D2A*,* IMPDH1*,* CNGA1*, and* EYS*.

### 3.3. Detection of Disease Causing Mutations in* RP1* Gene

After data filtering, the total number of putative disease causing variants was reduced by 99.3%. Thus, in total, filtering enriched the percentage of putative disease causing mutations from 0.7% (25/3339 variants) to 33.3% (9/25 variants) in the 7 subjects presented here (Table 2). These subjects exhibit* RP1* mutations in the last exon 4 that are predicted to lead to a premature stop codon. We found 9 pathogenic mutations in* RP1* among which one (p.Ser542^*^ in CIC00445) was already reported by Avila-Fernandez et al. [12] as a founder nonsense mutation in the Spanish population, responsible for 4.5% of arRCD. Although F303 is from French origin, we cannot exclude the possibility of a founder effect of p.Ser542^*^ in our cohort.

**Figure 3:**
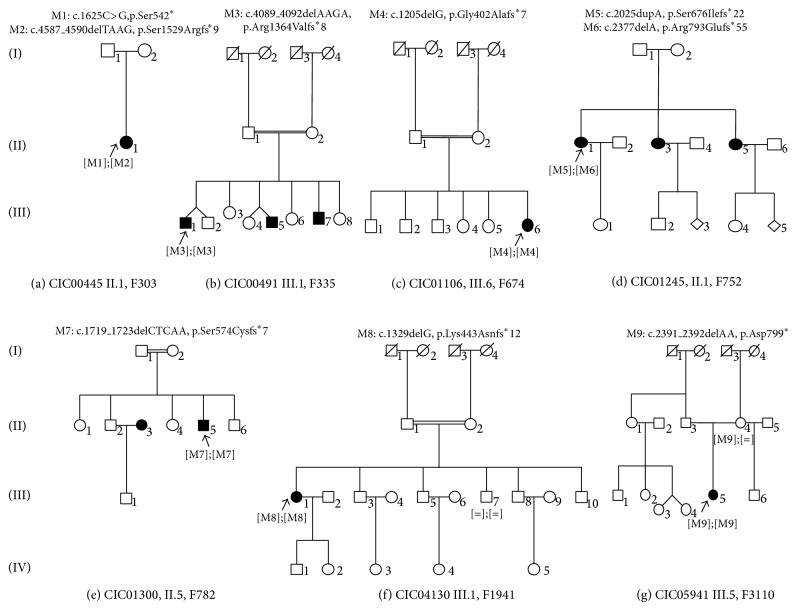
Pedigrees of seven families with* RP1* mutations underlying autosomal recessive rod-cone dystrophy. Affected and unaffected individuals are represented by shapes filled with black and white colors, respectively. Men and women are indicated by squares and circles, respectively. Index subjects are marked by ↖. Consanguinity is marked by a double horizontal line.

Patient family F303: II.1 (CIC00445) was found to carry compound heterozygous variants: a nonsense mutation c.1625C>G, leading to a predicted premature stop (p.Ser542^*^) and a deletion c.4587_4590delTAAG leading to a frameshift and a premature termination codon (p.Ser1529Argfs^*^9) (Table 2, Figure 3). Patient family F752: II.1 (CIC01245) was also found to carry compound heterozygous variants: a 1 bp duplication c.2025dupA leading to p.Ser676Ilefs^*^22 and a 1 bp deletion c.2377delA leading to p.Arg793Glufs^*^55 (Table 2).

Patients from family F335: III.1 (CIC00491), family F674: III.6 (CIC01106), family F782: II.5 (CIC01300), family F1941: III.1 (CIC04130), and family F3110: III.5 (CIC05941) were found to carry homozygous deletions c.4089_4092delAAGA leading to p.Arg1364Valfs^*^8; c.1205delG leading to p.Gly402Alafs^*^7; c.1719_1723delCTCAA leading to p.Ser574Cysfs^*^7; c.1329delG leading to p.Lys443Asnfs^*^12; and c.2391_2392delAA leading to p.Asp799^*^, resp.) (Table 2 and Figure 3). It is important to note that consanguinity was reported in families F335, F674, F782 and F1941.

All* RP1* mutations detected by NGS were further validated by Sanger sequencing. All variants cosegregated with the phenotype in available family members. Based on the previous findings, the measured prevalence of* RP1*-associated arRCD in this cohort is ≈2.5%.

## 4. Discussion

The current study further demonstrates the usefulness of NGS as a comprehensive genetic diagnostic tool for IRDs with further impact on patients counseling and participation for potential therapeutic trials. Our study applied to a large cohort of sporadic and autosomal recessive cases of RCD identifies 8 novel mutations in a gene not classically screened in arRCD by other methods such as Sanger sequencing or microarray analysis, outlining the interest of this massive parallel sequencing method. Consequently, a prevalence of* RP1* mutation in 2.5% of sporadic or arRCD cases in the European population is herein reported.

*RP1* is a 15 kb single copy gene clustering the small arm of the chromosome 8 (8q12.1). It encodes a 2506 amino acid protein having a molecular weight of 241 kDa containing a* Drosophila melanogaster* bifocal (BIF) (amino acid 486–635) and two doublecortin domains. Whereas the BIF domain helps to maintain the photoreceptor morphogenesis, doublecortin domains bind microtubules and regulate their polymerization [22]. Along with RP1L1 (Retinitis Pigmentosa 1-like 1, another retinal-specific protein), RP1 plays essential and synergistic roles in outer segment morphogenesis of rod photoreceptors [22].

To date, at least 50 mutations in* RP1* were identified in RCD [8, 12–20], the majority of which are located in its last exon (exon 4) and shown to be transmitted in an autosomal dominant mode of inheritance. Most of* RP1* disease causing variants represent nonsense mutations, deletions, or insertions. In mammalian genes, nonsense mutations lead to unstable mRNAs that are degraded by nonsense-mediated decay (NMD). However, exceptions might arise when premature stop codons occur in the last exon [37]. These variants are thought to abolish RP1 function by resulting in a truncated protein lacking important functional domains although still able to interact with some of its protein partner(s) [21]. The latter observation is supported by finding that* RP1* mutant mRNA is expressed in a human cell line carrying a homozygous p.Arg677^*^ mutation [21].

**Figure 4:**
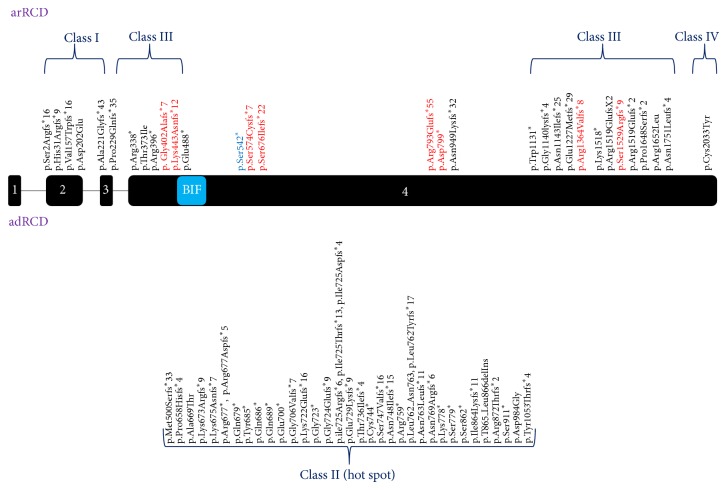
Schematic presentation of* RP1* disease causing mutations. Disease causing mutations were represented based on the classification by Chen and coworkers [13]. Mutations responsible for recessive arRCD were shown in the upper half, whereas mutations causing adRCD were shown in the lower half. p.Gly402Alafs^*^7, p.Lys443Asnfs^*^12, p.Arg1364Valfs^*^8, and p.Ser1529Argfs^*^9 belong to class III. Although p.Ser574Cysfs^*^7, p.Ser676Ilefs^*^22, p.Arg793Glufs^*^55, and p.Asp799^*^ are class II mutations, these variants do not cause adRCD but arRCD instead. Amino acid modifications shown in red and blue represent novel frameshift or nonsense mutations and the recurrent p.Ser542^*^ mutation respectively. Protein localization of p.Ser542^*^ was highlighted in blue as it marked a recurrent mutation. adRCD: autosomal dominant: rod-cone dystrophy, arRCD: autosomal recessive rod-cone dystrophy, BIF: drosophila melanogaster bifocal.

Based on Chen et al. [13],* RP1* truncating mutations leading to arRCD or adRCD can be divided into four distinct groups. Class I is composed of truncating mutations located in exons 2 and 3. These variants are sensitive to NMD and thus are considered as true loss-of-function alleles (Figure 4) [13]. Class II involves truncating mutations that are located in a spot between codons 500 and 1053 in exon 4 [13], the so called “*RP1* hot spot.” The “hot spot” variants tend to be insensitive to NMD process and thus result in a protein with a potential dominant negative effect leading to adRCD (Figure 4) [13]. Class III includes truncating mutations insensible to NMD located between codons 264 and 499 and between codons 1054 to 1751 in exon 4. These truncating proteins result in a loss of function leading to arRCD (Figure 4) [13]. Finally, class IV includes protein-truncating mutations near the 3′ end of the fourth exon (Figure 4) [13]. Most likely, the resulting proteins display only a minor loss of their C-terminal portion, preserving the majority of functional domains and keeping a residual activity. According to the classification of Chen et al. [13], p.Gly402Alafs^*^7, p.Lys443Asnfs^*^12, p.Arg1364Valfs^*^8, and p.Ser1529Argfs^*^9 belong to class III (Figure 4).

The predicted physiopathology for p.Ser542^*^, p.Ser574Cysfs^*^7, p.Ser676Ilefs^*^22, p.Arg793Glufs^*^55, and p.Asp799^*^ is more complex. According to Chen's classification, these frameshift deletions and nonsense mutations should belong to class II, previously only associated with adRCD. However, herein, they are causing presumably arRCD (Figure 4). To further confirm these findings, clinical and genetic testing of the reported unaffected parents should be done.

Based on the previous findings, we speculate that the classification by Chen and coworkers does not hold true for all mutations. Supporting this statement, Avila-Fernandez et al. [12] reported the same nonsense mutation (p.Ser542^*^) found in (F303: II.1 (CIC00445)) and located at the 5′ end of the “hot spot” to cause arRP in the Spanish population [12]. These observations are of interest as they point out for an implication of hot spot region for adRCD-*RP1* mutation also in case of arRCD. Future studies will need to clarify why some class II mutations lead to adRCD and others to arRCD.

Patients with arRCD and* RP1* mutations show a more severe disease than adRCD-RP1 mutant patients with macular atrophy in all our cases. This was first outlined by Lafont et al. [17]. When patients are presenting with late, severe disease, the diagnostic distinction between RCD, with initial rod involvement, and cone-rod dystrophy (CRD) with initial cone involvement is difficult. Of note is that one of the patients (CIC01300) in the present study was initially classified as possibly having severe CRD and his diagnosis was actually revisited after NGS results. This also outlines the interest of unbiased massive parallel sequencing methods for a more precise clinical diagnostic in case of end stage disease. This point will most likely become even more critical with the perspective of therapeutic trials.

### 4.1. Strength and Limitations

We estimate that 1% of our target regions were not covered. Partially uncovered exons are a real common issue when capturing the DNA sequences using commercially available probes; this bias might imply a loss of some candidate variants. However, we found that rate of 1% is very reasonable when compared with other NGS panels. In addition, in order to exclude the possibility of finding other candidate variants, we have sequenced by Sanger method the majority of these regions. Five of our patients carried homozygous* RP1* mutations. For four of the subjects carrying homozygous variants, namely CIC00491, F335; CIC01106, F674; CIC01300, F782 and CIC04130, F1941; co-segregation analysis needs to be done to confirm autosomal recessive inheritance but we do not have access to parent's DNA. CIC05941 was the only one not to report clear consanguinity in the family, and we cannot exclude the possibility of a large deletion on the second allele of* RP1* gene. Again, DNA of the father, not available for us, would be helpful to prove autosomal recessive inheritance and the homozygous state of the mutation.

In conclusion, we have reported 9 mutations in* RP1* of which 8 were novel causing arRCD [8, 12–20]. Interestingly, a prevalence of ≈2.5% points out for the necessity of sequencing* RP1* in sporadic and recessive cases of RCD. Further functional studies are needed to understand the impact of RP1 structure on its function at the molecular level; such a step would strengthen our knowledge in the physiology of retinal photoreceptors.

## Figures and Tables

**Table 1:** Clinical data of the 7 index patients with *RP1* recessive mutations.

Patient	Age at time of testing	Age of onset	Sex	Family history	Symptoms at time of diagnosis	BCVA OD/OS With refraction	Color vision (15 desaturated Hue)	Binocular kinetic visual field (III4e stimulus)	FF and mfERG	Fundus examination	FAF	Sd-OCT
F303: II.1 (CIC00445)	42	6	F	No other affected FM, from France.	Night blindness	Hand motion in both eyes	Impossible due to low vision	Reduced to peripheral islands of perception	Both undetectable	Pale optic disc narrowed blood vessels, macular atrophic changes, and optic nerve drusen	Hypoautofluorescence in the macular region	Thinning of outer retina in the macular region

F335: III.1 (CIC00491)	36	3	M	Two other brothers affected; parents first cousins	Night blindness and rapid decreased vision	LP in both eyes	Impossible due to low vision	Impossible due to low vision	Both undetectable	Widespread RPE changes and retinal atrophy in both the periphery and the macular area	Widespread loss of FAF	Widespread thinning of outer retina

F674: III.6 (CIC01106)	25	19	F	Parents first cousins from Turkey, one female and male cousins affected also from a consanguineous union	Night blindness and decreased vision	HM −3 (−1) 0° 20/160 −3 (−0.50) 0°	Dyschromatopsia with no specific axis	Reduced to 5 central degrees	Both undetectable	Well-colored optic disc and no narrowing of retinal vessels; RPE changes in the periphery and macular atrophic changes	Hypoautofluorescence in the macular region and outside the vascular arcades	Thinning of outer retina in the macular region

F752: II.1 (CIC01245)	31	Early teens	F	Two sisters affected	Night blindness	20/63 plano (−3) 180° 20/50 plano (−1.75) 180°	Deutan defect on both eyes	Reduced to 10° × 20°	Both undetectable	Pale optic disc head, narrowed retinal vessels, and RPE changes in the periphery with some macular atrophic changes	Hypoautofluorescence in the macular region and outside the vascular arcades	Thinning of outer retina in the macular region

F782 II.5 (CIC01300)	27	9	M	Parents from Algeria, first cousins	Night blindness and decreased vision	20/50 −9.25 (−2.50) 15° 20/50 −9 (−1.75) 100°	—	Reduced to the 10 central degrees	Both undetectable	Mild optic disc pallor, atrophic macular changes, and peripheral pigment deposits	Hypoautofluorescence in the macular region	Thinning of outer retina in the macular

F1941: III.1** (**CIC04130)	30	childhood	F	Parents from Algeria, first cousins	Night blindness	20/100 −4.25 (−1.25) 150° 20/80 −4.25 (−1.25) 150°	Normal at the saturated test	Reduced to the 10 central degrees	Both undetectable	Well-colored optic disc but narrowed retinal vessels; RPE changes in the periphery and macular atrophic changes	Hypoautofluorescence in the macular region and outside the vascular arcades	Thinning of outer retina in the macular region

F3110: III.5 (CIC05941)	27	5	F	One cousin on mother side may have RCD	Night blindness and decreased vision	20/125 +2 (−2) 95° 20/125 +1.75 (−2) 70°c	Dyschromatopsia with no specific axis	Reduced to the 10 central degrees	Both undetectable	Pale optic disc, narrowed retinal vessels, and RPE changes in the periphery with some macular atrophic changes	Hypoautofluorescence in the macular region and outside the vascular arcades	Thinning of outer retina in the macular region

F: female, FM: family member, M: male, BCVA: best corrected visual acuity; OD: ocula dextra (right eye); OS: ocula sinistra (left eye); FF and mfERG: full-field and multifocal ERG; FAF: fundus autofluorescence; Sd-OCT: spectral domain optical coherence tomography; RPE: retinal pigment epithelium; LP: light perception; HM: hand motion.

**Table 2:** List of mutations detected by next generation sequencing after applying relevant filters.

Patient	Gene	Exon	Allele state	Nucleotide exchange	Protein effect	rs ID	Conservation	Polyohen 2	SIFT	Pathogenicity	Note
F303: II.1 (CIC00445)	*NPHP4 *	12	HTZ	A>G	p.Ser481Asn	no	N C	B	T	Neutral	
	***RP1***	**4**	**HTZ**	**c.1625C>G**	**p.Ser542** ^*^	—	—	—	—	**Disease causing**	**R M** [12]
	***RP1***	**4**	**HTZ**	**c.4587_4590delTAAG**	**p.Ser1529Argfs** ^*^ **9**	—	—	—	—	**Disease causing**	**N M**

F335: III.1 (CIC00491)	*PROM1 *	4	HTZ	T>C	p.Ile178Val	—	**N C**	B	T	Neutral	
	*GPR98 *	29	HTZ	G>A	p.Arg2128Gln	rs149390094	**N C**	B	T	Neutral	
	***RP1***	**4**	**HMZ**	**c.4089_4092delAAGA**	**p.Arg1364Valfs** ^*^ **8**	—	—	—	—	**Disease causing**	**N M**

F674: III.6 (CIC01106)	*USH2A *	39	HTZ	T>G	p.Ser2450Arg	No	**H C**	P D	D	**Probably disease causing**	
	***RP1***	**4**	**HMZ**	**c.1205delG**	**p.Gly402Alafs** ^*^ **7**	—	—	—	—	**Disease causing**	**N M**

F752: II.1 (CIC01245)	*USH1C *	17	HTZ	G>A	p.Arg477Trp	TMP_ESP_11_17532053	H C	P D	D	Probably disease causing	
	*PDE6B *	10	HTZ	T>C	p.Thr432Ile	—	H C	B	T	Uncertain pathogenicity	
	***RP1***	**4**	**HTZ**	**c.2025dupA**	**p.Ser676Ilefs** ^*^ **22**	—	—	—	—	**Disease causing**	**N M**
	***RP1***	**4**	**HTZ**	**c.2377delA**	**p.Arg793Glufs** ^*^ **55**	—	—	—	—	**Disease causing**	**N M**

F782: II.5 (CIC01300)	***RP1***	**4**	**HMZ**	**c.1719_1723delCTCAA**	**p.Ser574Cysfs** ^*^ **7**	—	—	—	—	**Disease causing**	**N M**
	*TULP1 *	5	HMZ	c.395_418dup	p.Asp124_Glu131del	rs63749128	—	—	—	Neutral	

F1941: III.1 (CIC04130)	*PCDH15 *	33	HTZ	C>T	p.Arg1889His	rs145851144	N C	B	T	Neutral	
	*C2orf71 *	1	HTZ	C>A	p.Arg656Ser	rs201980758	N C	B	T	Neutral	
	*CACNA2D4 *	Exon37-Intron 37	HTZ	C>T	—	rs80092457	N C	—	—	Neutral	
	***RP1***	**4**	**HMZ**	**c.1329delG**	**p.Lys443Asnfs** ^*^ **12**	—	—	—	—	**Disease causing**	**N M**

F3110: III.5 (CIC05941)	*EYS *	6	HTZ	C>T	p.Ser326Asn	rs112822256	N C	B	T	Neutral	
	*MERTK *	8	HTZ	C>G	p.Arg421Trp	rs138908058	N C	B	D	Neutral	
	*PRPF6 *	21	HTZ	A>G	p.Val915Met	rs139778757	M C	P D	D	Uncertain pathogenicity	
	*TULP1 *	14	HTZ	G>A	p.Ala496Thr	rs141980901	M C	B	D	Neutral	
	*EYS *	26	HTZ	G>A	p.Lys1365Glu	rs16895519	N C	B	D	Neutral	
	*MERTK *	18	HTZ	G>C	p.Glu823Gln	rs55924349	M C	B	D	Neutral	
	***RP1***	**4**	**HMZ**	**c.2391_2392delAA**	**p.Asp799** ^*^	—	—	—	—	**Disease causing**	**N M**

Probably disease causing mutations are highlighted in bold.

B: benign, HMZ: homozygous, HTZ: heterozygous, M C: marginally conserved, N C: not conserved, N M: novel mutation, R M: recurrent mutation, T: tolerated, P.D: possibly damaging.
